# Thymoquinone Defeats Diabetes-Induced Testicular Damage in Rats Targeting Antioxidant, Inflammatory and Aromatase Expression

**DOI:** 10.3390/ijms18050919

**Published:** 2017-04-27

**Authors:** Mustafa S. Atta, Essam A. Almadaly, Ali H. El-Far, Rasha M. Saleh, Doaa H. Assar, Soad K. Al Jaouni, Shaker A. Mousa

**Affiliations:** 1 Department of Physiology, Faculty of Veterinary Medicine, Kafrelsheikh University, Kafrelsheikh 33516, Egypt; mostafa.ataa@vet.kfs.edu.eg; 2 Department of Theriogenology, Faculty of Veterinary Medicine, Kafrelsheikh University, Kafrelsheikh 33516, Egypt; essam.mostafa@vet.kfs.edu.eg; 3 Department of Biochemistry, Faculty of Veterinary Medicine, Damanhour University, Damanhour 22511, Egypt; ali.elfar@damanhour.edu.eg; 4 Department of Animal Physiology, Faculty of Veterinary Medicine, Mansoura University, Mansoura 35516, Egypt; rasha_physiology@yahoo.com; 5 Department of Clinical Pathology, Faculty of Veterinary Medicine, Kafrelsheikh University, Kafrelsheikh 33516, Egypt; doaa.abdelhady1@vet.kfs.edu.eg; 6 Hematology/Pediatric Oncology, King Abdulaziz University Hospital and Scientific Chair of Yousef Abdullatif Jameel of Prophetic Medicine Application, Faculty of Medicine, King Abdulaziz University, Jeddah 21589, Saudi Arabia; saljaouni@kau.edu.sa; 7 Pharmaceutical Research Institute, Albany College of Pharmacy and Health Sciences, Rensselaer, NY 12144, USA

**Keywords:** thymoquinone, testicular deterioration, oxidative stress, iNOS, NF-κB, aromatase, diabetic rat

## Abstract

Antioxidants have valuable effects on the process of spermatogenesis, particularly with diabetes mellitus (DM). Therefore, the present study investigated the impact and the intracellular mechanisms by which thymoquinone (TQ) works against diabetes-induced testicular deteriorations in rats. Wistar male rats (*n* = 60) were randomly allocated into four groups; Control, Diabetic (streptozotocin (STZ)-treated rats where diabetes was induced by intraperitoneal injection of STZ, 65 mg/kg), Diabetic + TQ (diabetic rats treated with TQ (50 mg/kg) orally once daily), and TQ (non-diabetic rats treated with TQ) for 12 weeks. Results revealed that TQ significantly improved the sperm parameters with a reduction in nitric oxide (NO) and malondialdehyde (MDA) levels in testicular tissue. Also, it increased testicular reduced glutathione (GSH) levels and superoxide dismutase (SOD) activity. Interestingly, TQ induced downregulation of testicular inducible nitric oxide synthase (iNOS) and nuclear factor kappa-B (NF-κB) and significantly upregulated the aromatase protein expression levels in testicles in comparison with the diabetic rats. In conclusion, TQ treatment exerted a protective effect against reproductive dysfunction induced by diabetes not only through its powerful antioxidant and hypoglycemic effects but also through its downregulation of testicular iNOS and NF-κB along with upregulation of aromatase expression levels in diabetic rats.

## 1. Introduction

Diabetes mellitus (DM) is a metabolic disorder accompanied by hyperglycemia due to inadequate insulin secretion or insulin resistance [1]. Spermatogenesis is associated with many diseases, for example, DM, coronary heart diseases, and chronic liver diseases [2]. DM has adverse effects on fertility of diabetic patients and animals; for instance, it causes a decrease in testicular and body weights and abnormal spermatogenesis with sperm deformities [3]. Sperm cells encompass a high amount of specific lipid composition such as polyunsaturated fatty acid, plasmalogen, and sphingomyelin. This high amount of polyunsaturated fatty acid in the absence of antioxidant mechanism renders mammalian spermatozoa more vulnerable to oxidative damage [4], where DM is related to the overproduction of cellular reactive oxygen species (ROS) that instigate testicular injuries [5]. Thus, it hurts fertility, which might be implicated in lower fertility in males [6].

Thymoquinone (TQ; 2-isopropyl-5-methyl-1,4-benzoquinone) is the active ingredient of *Nigella sativa* seeds with various pharmacological potentials including antioxidant, anticancer, antidiabetic, anti-inflammatory, and antimicrobial activities [7,8]. Several studies showed that TQ significantly improved semen quality and reproductive characteristics of mice exposed to heat stress [9], re-established spermatogenesis after testicular injury in rats [10], and controlled methotrexate-induced testicular damage in mice [11] and rats [12]. Additionally, TQ protects rats from the testicular damage induced by cadmium exposure through its antioxidant and anti-inflammatory activities [13]. Therefore, the present study was conducted to evaluate the ameliorative effect of TQ on diabetic male rats concerning the intracellular pathway by which it may be useful to decrease diabetic infertility.

## 2. Results

**Figure 1:**
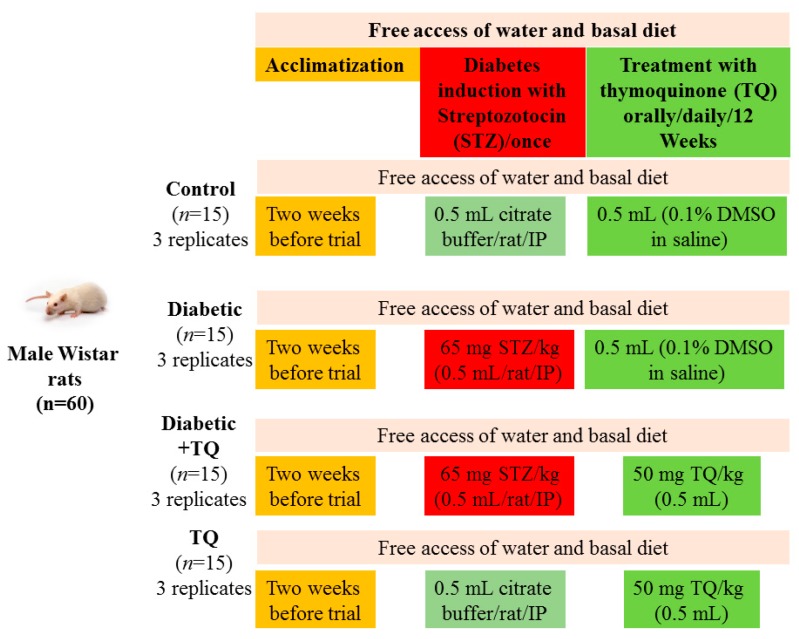
Experimental design.

The overall experimental design including animal groups and treatments is shown in Figure 1.

### 2.1. Survival Percentages

**Figure 2:**
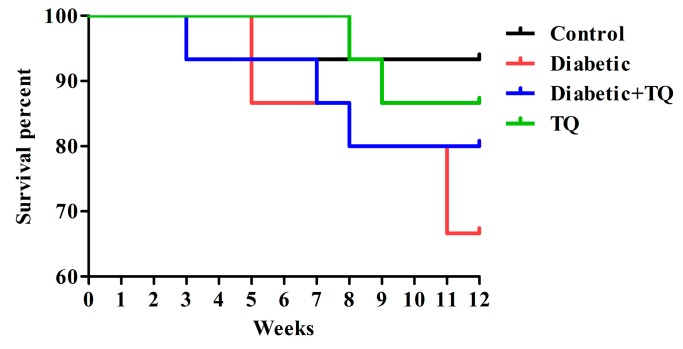
Survival percentages in groups: Control (93.33%), Diabetic (66.67%), Diabetic + TQ (80%), and TQ (86.67%). TQ; thymoquinone.

The number of animal deaths was recorded each week during the trial period where the control group elucidates the survival rate (93.33%); one rat died due to drug perfusion to the esophagus by stomach tube at the 5th week. Survival percentages for the groups were as follows: Diabetic: 66.67%; Diabetic + TQ: 80%; TQ: 86.67% (Figure 2).

### 2.2. Body Weight and Accessory Glands Weight

**Figure 3:**
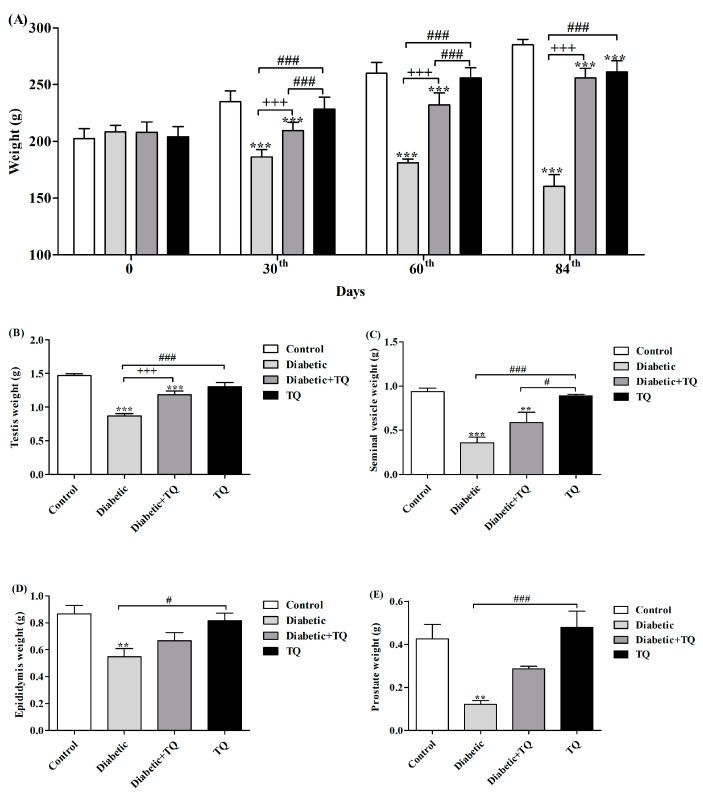
Body weight (**A**), relative weight of testicles (**B**), seminal vesicle (**C**), epididymis (**D**), prostate gland (**E**) in Control, Diabetic, Diabetic + TQ, and TQ groups. ** *p* < 0.01 and *** *p* < 0.001 vs. Control. ^+++^ *p* < 0.001 vs. Diabetic. ^#^ *p* < 0.05 and ^###^ *p* < 0.001 vs. TQ. TQ; thymoquinone.

At the end of the experiment (84th day), the body weight of diabetic rats was significantly decreased (*p* < 0.05) when compared with control and TQ-treated diabetic groups (Figure 3A), and the relative body weight of testicles, prostate, seminal vesicles, and epididymis significantly decreased (*p* < 0.05) when compared with control and TQ-treated diabetic groups (Figure 3B–E). TQ supplementation to the normal rats had no significant effect (*p* > 0.05) on its body weight.

### 2.3. Serum Glucose, Glycated Hemoglobin, and Insulin

Oral supplementation of TQ to STZ-induced diabetic rats ameliorated the higher blood glucose level (*p* < 0.05) as stated for the diabetic group. Diabetic rats showed a significant (*p* < 0.05) increase in hemoglobin A1c (HbA1c) percentage in comparison with control and other groups (Table 1). Furthermore, there were no significant differences between TQ-treated rats and the control group.

### 2.4. Sperm Parameters

**Figure 4:**
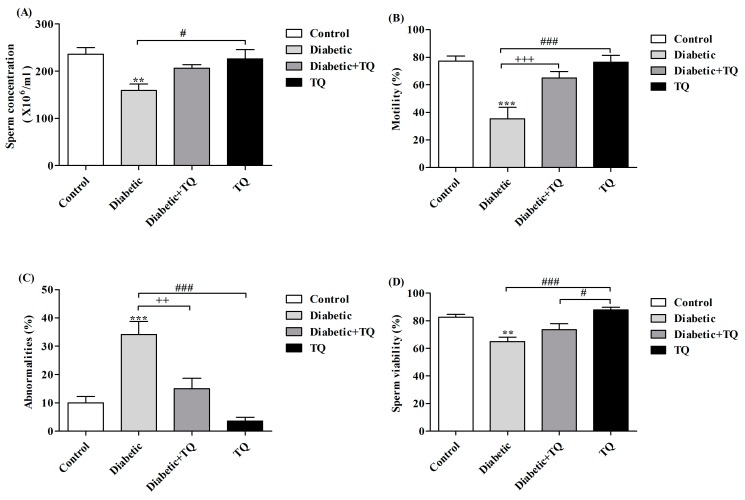
Sperm cell concentration (**A**), Motility % (**B**), Abnormalities (**C**), and Viability (**D**) in Control, Diabetic, Diabetic + TQ, and TQ groups. ** *p* < 0.01 and *** *p* < 0.001 vs. Control. ^++^ *p* < 0.01, ^+++^ *p* < 0.001 vs. Diabetic. ^#^ *p* < 0.05, and ^###^ *p* < 0.001 vs. TQ. TQ: thymoquinone.

Results in Figure 4 show the semen picture of diabetic rats, which revealed that diabetic rats had lower sperm concentration, motility, and viability, with a higher percentage of sperm abnormalities. On the contrary, TQ-treated diabetic rats had higher sperm concentration, motility, and viability and lower abnormalities in comparison with control and diabetic rats. Rats in the TQ group had non-significant improvement in sperm parameters in comparison to the control group.

### 2.5. Testosterone and Estradiol

The results showed significant decreases (*p* < 0.05) in testicular acid phosphatase (ACP) and improvement in alkaline phosphatase (ALP) as determined in the testicular homogenate of diabetic rats as compared with control and TQ-treated groups, which had restored ACP and ALP activities as in the control, while in the TQ group, TQ kept the ACP and ALP near their levels in Control (Table 2).

**Figure 5:**
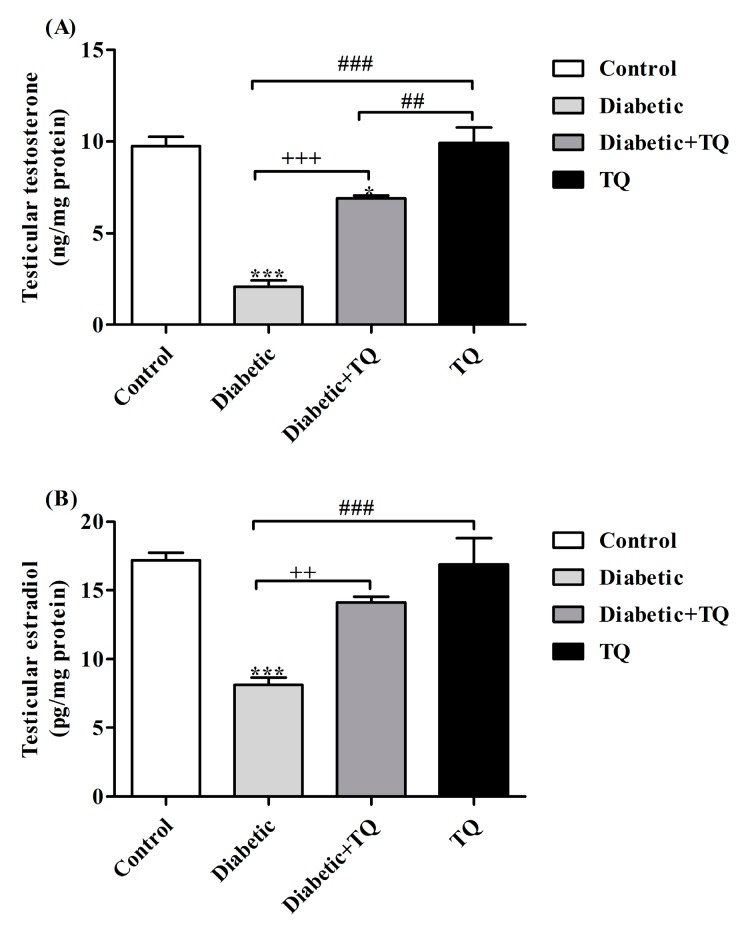

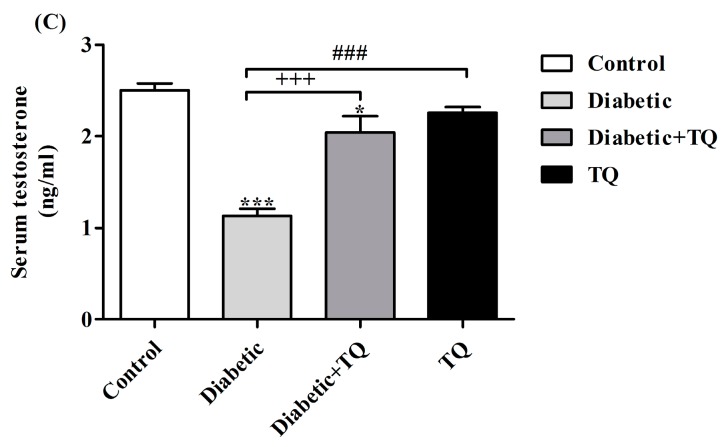
Levels of testicular testosterone (**A**) and estradiol (**B**) and serum testosterone (**C**) in Control, Diabetic, Diabetic + TQ, and TQ groups. * *p* < 0.05 and *** *p* < 0.001 vs. Control. ^++^ *p* < 0.01, ^+++^ *p* < 0.001 vs. Diabetic. ^##^ *p* < 0.01, and ^###^ *p* < 0.001 vs. TQ. TQ; thymoquinone.

The data illustrated in Figure 5 showed a significant decrease (*p* < 0.05) in serum testosterone concentration, testicular testosterone, and estradiol levels in diabetic rats compared with other groups (*p* < 0.05). However, the TQ-treated group showed greater testicular testosterone and estradiol levels as well as serum testosterone concentrations compared with diabetic rats.

### 2.6. Testicular Histopathology and Spermatogenesis

Control and TQ-treated groups exhibited normal testicular construction and spermatogenesis with active Leydig cells, whereas diabetic rats showed degeneration of seminiferous tubules and sloughing of the germinal epithelium. The deleterious effect of diabetes was improved by TQ, which was proven by restoration of seminiferous tubules with active Leydig cells. Regarding spermatogenesis, Johnsen scores were 9.86, 4.97, 8.71, and 9.77 for Control, Diabetic, Diabetic + TQ, and TQ, respectively. There was a significant improvement in the TQ-treated group in comparison with the diabetic group (Table 2).

### 2.7. Testicular Oxidative Stress and Antioxidant Status

The effects of TQ on lipid peroxidation and the antioxidant status of testicles in diabetic rats were investigated, and the antioxidant capacities were found to have significant differences (*p* < 0.05) (Table 2). Conversely, diabetic rats showed significantly (*p* < 0.05) higher testicular malondialdehyde (MDA) concentration compared with the control group. Testicular GSH levels in STZ-treated rats were significantly (*p* < 0.05) lowered (Table 2). Oral supplementation of TQ significantly (*p* < 0.05) restored the testicular GSH drop in Diabetic + TQ rats. Testicular superoxide dismutase (SOD) activities were significantly reduced (*p* < 0.05) in diabetic rats when compared with the control. Additionally, TQ administration to diabetic rats significantly improved testicular SOD activities in relation to diabetic rats. STZ-induced diabetic rats had significant increases (*p* < 0.05) in testicular NO levels in comparison with the control. Oral supplementation of TQ to diabetic rats maintained normal NO level compared to control values (Table 2).

### 2.8. INOS, NF-κB-p65, and Aromatase Expressions in Testicular Homogenate

**Figure 6:**
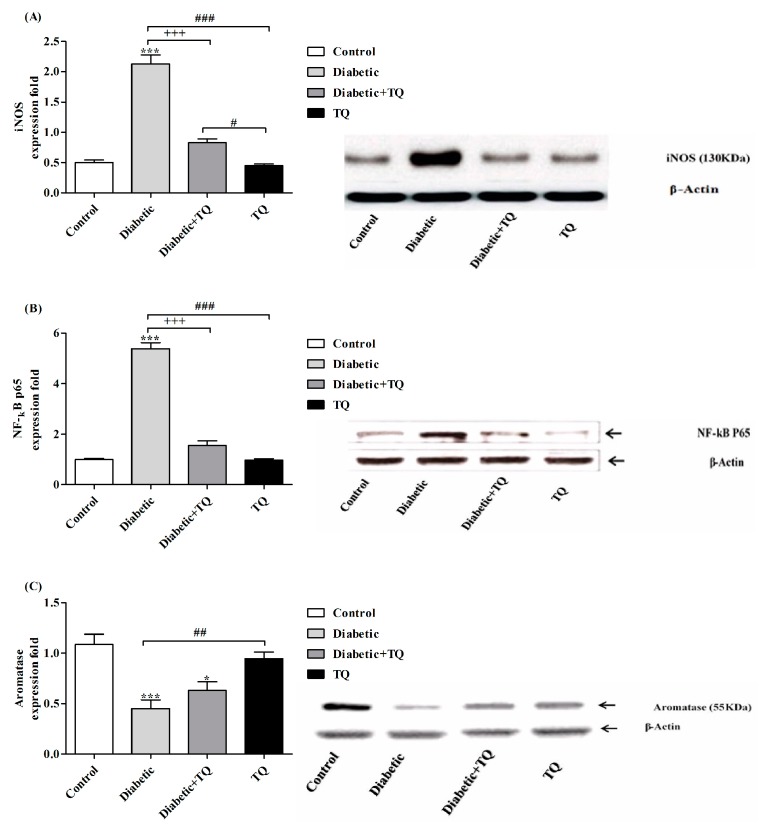
Western blotting analysis of iNOS (**A**), NF-κB-p65 (**B**), and Aromatase (**C**) in Control, Diabetic, Diabetic + TQ, and TQ groups. * *p* < 0.05 and *** *p* < 0.001 vs. Control. ^+++^ *p* < 0.001 vs. Diabetic. ^#^ *p* < 0.05, ^##^ *p* < 0.01 and ^###^ *p* < 0.001 vs. TQ. TQ; thymoquinone.

The testicular iNOS protein expressions were significantly higher (*p* < 0.05) in the diabetic group in comparison with the other groups, as presented in Figure 6A. The TQ-treated diabetic group showed significantly decreased (*p* < 0.05) iNOS protein expressions and they returned to the same levels as the control group. Testicular NF-κB-p65 expression was more activated in the diabetic rat than in both control and TQ-treated groups. Oral supplementation of TQ to the diabetic rats caused a significant reduction (*p* < 0.05) of NF-κB-p65 expression (Figure 6B). In testicular tissue, aromatase immuno-reactivity notably decreased at 12 weeks in the diabetic group compared with the control and TQ groups. The Diabetic + TQ group showed a greater significant (*p* < 0.05) improvement in aromatase expression compared with the diabetic group (Figure 6C).

### 2.9. Histopathology

**Figure 7:**
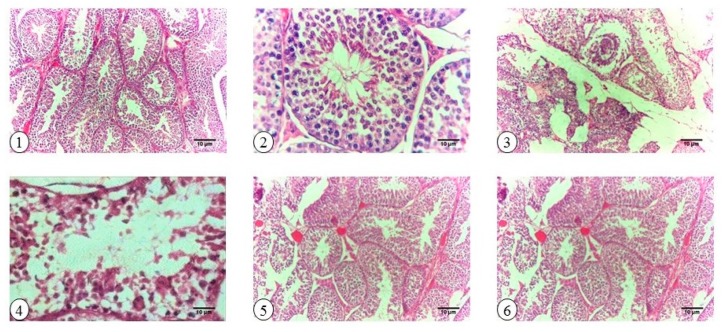
Cross sections of histopathological examination of testicular samples in Control, Diabetic, Diabetic + TQ, and TQ-treated groups stained with H&E. (**1**) Control group rats show intact seminiferous tubules and regular spermatogenesis and active Leydig cell, 100×. (**2**) Control group rats show free spermatozoa in the lumen of the seminiferous tubules, regular spermatogenesis, and active Leydig cell, 400×. (**3**) Diabetic rats show apparent degeneration of the seminiferous tubules, sperm giant cell, and pyknotic spermatocytes, 100×. (**4**) Diabetic rats show sloughing of the germinal epithelium, lysis of the spermatocytes, and necrosis of the spermatids, 400×. (**5**) TQ-treated diabetic rats show resuscitation of the seminiferous tubules’ enhancement (100×) in the tubular basal compartment with the presence of spermatogonia. (**6**) TQ rats show normal seminiferous tubules, 100×. TQ; thymoquinone.

Results obtained from histopathological examination indicated that the testicles of diabetic rats had marked degeneration of seminiferous tubules, sperm giant cell, and pyknotic spermatocytes in addition to sloughing of the germinal epithelium, lysis of the spermatocytes, and necrosis of the spermatids. On the contrary, samples from TQ-treated rats showed normal seminiferous tubules (Figure 7).

## 3. Discussion

Our data revealed that the rats’ body weight and relative weight of accessory glands decreased during the course of diabetes; these results were consistent with those obtained by Ozdemir et al. [14], who attributed the reduction of body weight to the breakdown of tissue protein in the diabetic group. Diabetes causes a significant increment in skeletal muscle catabolism along with a decline in protein synthesis [15].

Relative body weights of testicles, prostate, seminal vesicle, and epididymis were decreased in diabetic rats and that may be attributed to the testicular deterioration and decline of the epididymis weight. In addition, testicles and other reproductive tissues depend upon testosterone, which motivates growth and secretary action of the reproductive organs [16]. Additionally, Soudamani et al. [17] have shown that the atrophic alterations in the epididymis are due to reduced tubular diameter, volume, and surface density in diabetic rats. Diabetic rats showed hyperglycemia and decreased serum insulin level in the current study; this finding is in harmony with results from Ballester et al. who found that diabetic rats displayed a reduction in the concentration of serum insulin, testosterone, follicle-stimulating hormone (FSH), and luteinizing hormone (LH) [18]. Decreased insulin levels reflected a sharp weakening of spermatogenesis, consistent with results reported by Bruening [19].

Determination of HbA1c level is a pivotal biomarker for diagnosis and prognosis of diabetes and its complications. The current study showed that diabetic rats had a significant increase in HbA1c percentage when matched with control and TQ-treated groups and as proved in previous studies by Abdelmeguid et al. [7] and Meral et al. [20], who stated that *Nigella sativa* oil and TQ reduced the blood glucose and HbA1c and increased insulin levels. The noted hypoglycemia may be due to the improvement of β-cell ultrastructure, thus directing improved insulin levels, which aids glucose uptake with normoglycemia. Additionally, TQ suppresses the endogenous glucose liberation either by glycogenolysis or gluconeogenesis in liver and muscles [21]. In the TQ group, serum glucose, HbA1c, and insulin levels were non-significantly different from the control because TQ is a normoglycemic compound that induces hypoglycemia in hyperglycemic state.

Hyperglycemia of DM raises the level of ROS that produces DNA destruction in testis and a major reduction in sperm parameters such as sperm motility, count, and viability [5]. On the contrary, TQ markedly improves the sperm parameters by scavenging of the ROS and activation of testicular enzymatic antioxidant status. Concerning the sperm cell count, it is the most critical assessment value for spermatogenesis, and it is highly associated with fertility. Diabetic rats exhibit a marked decrease in sperm concentration, live sperm percentage, and increased sperm abnormalities. These findings were recognized by Scarano et al. [22], who confirmed that diabetic rats had pronounced reduction in sperm quantity and quality due to associated oxidative injuries.

Testicular testosterone is crucial for spermatogenesis and high testosterone level is essential for the normal physiology of seminiferous tubules [23], while estradiol is biosynthesized by the action of aromatase enzyme mainly in the Leydig cells. Estradiol functions to control apoptosis of male sperm cells [24]. In the present study, testicular testosterone and estradiol were markedly decreased in diabetic rats, as also found by Farrell et al. [25]. Besides, we found the mean Johnsen score value was 4.97 in the diabetic rats and it significantly improved in the Diabetic + TQ group. This finding explores the protective effect of TQ on spermatogenesis. Ballester et al. [18] suggested that the low level of testosterone in diabetic rats may be related to the decrease in Leydig cells or in androgen biosynthesis.

Diabetes provoked a state of oxidative injury that was proved by an increase in levels of testicular MDA and a reduction in SOD activity. Bauche et al. [26] reported a noticeable increase in lipid peroxidation product, MDA, levels in diabetic rats. The oxidative stress caused by diabetes was shown by a drastic decrease in spermatogenesis values [27]. TQ has protected diabetic rats against the harmful effects of DM through a marked enhancement in testicular antioxidant enzymes [28]. Further, TQ suppresses the cyclooxygenase-2 enzyme expression and lipid peroxidation and raises SOD activities in diabetic rats [29]. 

The oxidative damage in diabetic rats led to the liberation of ALP and ACP to the blood and increased their levels as a biomarker of testicular injuries. This leakage in testicular ALP and ACP was counteracted by TQ treatment. Therefore, the testicular levels of both ALP and ACP were significantly decreased in response to oxidative injuries induced in the diabetic group. Conversely, TQ had been protecting the testicular injuries and kept the levels of ALP and ACP around the normal activities as in the control group. The activity of testicular ACP was decreased in malathion-treated due to damage in testicular tissue [30]. The altered enzyme activities may be coming from the damaged seminiferous epithelium and the function of the Sertoli cell. ALP is the testicular biomarker; decrease in ALP level is directly associated with the atrophy of seminiferous tubules, the absence of the spermatozoa in the lumen, the loss of spermatogenic cell layers, and the degeneration of Leydig cell in Tributyltin-treated hamster [31].

DM is accompanied by the overexpression of iNOS and NF-κB-p65 with a simultaneous rise in the testicular NO. It is well known that NO and pro-inflammatory mediators elicit reproductive dysfunction by generating testicular injuries that lead to testicular atrophy and apoptosis [32,33]. Results of the current study are consistent with diabetic rats exhibiting upregulation of both iNOS and NF-κB-p65 protein expression and a drastic increase in NO levels. Upregulation of the iNOS expression enhances the creation of an abundance of NO. High levels of NF-κB and iNOS are main biomarkers of inflammatory responses [34]. Results revealed significant increases in the testicular NO levels in Diabetic + TQ group along with the downregulation of iNOS expression. The previous study by Kanter found that TQ treatment to chronic toluene exposure in rats markedly decreased the iNOS expression in the Leydig cells [10]. Further, TQ reduced the expression of TNF-α, iNOS, and IL-1β and attenuated the overexpressed NF-κB. Additionally, the antioxidant enzyme activities of the rat liver were markedly increased [35].

Aromatase (EC 1.14.14.1) is a member of the cytochrome P450 family involved in development and reproduction [36]. It has been noticed that aromatase deficiency distorts glucose and lipid metabolism [37]. Nevertheless, aromatase expression levels in testicular tissues of diabetic rats were markedly decreased, as reported by Burul-Bozkurt et al. [38]. In the same manner, aromatase has essential physiological roles in male fertility [39]. Therefore, the decreased sperm quality has been recorded in men who have mutations in aromatase gene [40]; decreased sperm quality and quantity has also been found in diabetic males. Overall, the reduced aromatase levels might be one of the critical mechanisms responsible for male reproduction dysfunctions in DM [5].

## 4. Materials and Methods

### 4.1. Chemicals 

Streptozotocin (STZ), dimethyl sulfoxide (DMSO), ethylenediaminetetraacetic acid (EDTA), TQ, glucose, 0.1 M citrate buffer, phosphate buffered saline, and sodium chloride solution (0.9%) were purchased from Sigma-Aldrich (Sigma Chemical Co., St. Louis, MO, USA). Antibodies against iNOS, NF-κB, aromatase and β-actin were purchased from Santa Cruz Biotechnology, Inc. (Santa Cruz, CA, USA).

### 4.2. Animals

All experiments performed were approved by the ethics committee in Faculty of Veterinary Medicine, Kafrelsheikh University, Egypt, ethical issue number KVM016/2014 (April 2014). Sixty male Wistar rats weighing 180–200 g were reared in the Department of Physiology, Faculty of Veterinary Medicine, Kafrelsheikh University Egypt and housed in well-ventilated plastic cages. They received the diet and water ad libitum. The composition of the experimental diet is stated in Table 3. All rats were maintained on a 12:12 h light/dark cycle. Rats were kept untreated for two weeks for acclimatization before treatment.

### 4.3. Induction of Diabetes

Immediately after STZ was dissolved in 0.1 M citrate buffer of pH 4.5, rats were injected intraperitoneally (IP) with STZ in a dose of 65 mg/kg body weight (0.5 mL/rat) after fasting for 16 h [41]. The STZ-injected rats were given 5% glucose (10 mL/rat) for 24 h to avoid initial hypoglycemic death. Control rats were injected with 0.5 mL citrate buffer per rat. One week later, blood samples were collected from the tail vein to determine the serum glucose level using a glucometer (IME-DC GmbH Co., Hof, Germany). Rats with serum glucose more than 250 mg/dL were considered diabetic as stated by Kaplanoglu et al. [42].

### 4.4. Preparation of Thymoquinone

Light-proof and cooled (4 °C) TQ was dissolved initially in DMSO (0.1%), followed by the addition of normal saline (0.9% NaCl). The solution was administered at a dose of 50 mg/kg body weight once daily by using gastric gavage, for 12 successive weeks [43].

### 4.5. Experimental Design

The overall experimental design including animal groups and treatments is shown in Figure 1. Rats were randomly assigned into four groups (*n* = 15), and each group was subdivided into three replicates (*n* = 5). The treatment schedule was as follows: control rats were injected IP once with 0.5 mL of 0.1 M citrate buffer. Additionally, diabetic rats were injected IP once with 0.5 mL of 65 mg/kg body weight STZ for induction of diabetes. For the Diabetic + TQ group, rats were orally treated with TQ at a dose of 50 mg/kg body weight once daily [43,44], and TQ rats were given 0.5 mL of TQ 50 mg/kg body weight once daily, orally by the aid of gastric gavage, for 12 successive weeks. Additionally, all non-TQ groups received 0.5 mL of normal saline containing DMSO (0.1%) by gavage daily. After 12 weeks, the rats were sacrificed, under anesthesia with intravenous injection of sodium pentobarbital (30 mg/kg), for proper sampling and subsequent analysis. 

### 4.6. Sampling

After 84 days from STZ treatment, rats were euthanized by cervical decapitation and two blood samples were collected from each rat. One sample was collected into a tube containing ethylenediaminetetraacetic acid (EDTA) for hemoglobin A1c (HbA1c) assessment. The second sample was allowed to coagulate and then centrifuged at 1435× *g* for 5 min. The clear sera were collected and subjected to biochemical analysis.

For each rat, testicles were excised, cleaned, and washed in cold saline. The testicles and accessory sex organs, seminal vesicles, prostates, and epididymis were dissected and individually weighed. Part of the left testis of each rat was homogenized in cold phosphate buffer saline (PBS). The homogenates were centrifuged at 3000× *g* for 10 min at 4 °C. The collected supernatants were stored at −20 °C. The other specimen from the left testis was fixed with 10% formalin solution for histopathological examination, and the other was kept frozen at −80 °C for a Western blotting assay.

### 4.7. Semen Evaluation

Cauda epididymis was cut into small pieces and incised to liberate spermatozoa on a warm, clean glass slide. Exactly 2 μL from liberated spermatozoa were mixed with 20 μL of 2.9% sodium citrate and coverslipped. The percentage of motile sperm was individually evaluated at 400× magnification in at least two microscopic fields [45]. Sperm count was estimated using a hemocytometer and viewed under a magnification of 40×. Seminal smears were stained with eosin-nigrosine stain and examined to determine the proportion of viable and abnormal spermatozoa [46].

### 4.8. Assessment of Spermatogenesis

Spermatogenesis indexes were done using Johnsen scores to sort spermatogenesis at 400× magnification power. To evaluate the quality of the specimen, we used a slightly modified Johnsen score count [47]. Microscopically, the spermatogenesis values were classified as 1, for Sertoli cells only; 2, for spermatogonia only; 3 and 4, for no further than primary spermatocytes (<10 and 10–30 spermatocytes per view, respectively); 5, 6, and 7, for no further than round spermatids (<10, 10–40, and >40 spermatids per view, respectively); 8, 9, and 10, for maturation phase spermatids (<20, 20–50 and >50 spermatids per view, respectively). 

### 4.9. Biochemical Assay

Serum glucose level was assessed with an enzymatic glucose kit following the method of Trinder [48]. The HbA1c percentage was estimated using Helena GLYCO-Tek affinity column method (Helena Laboratories, Beaumont, TX, USA) [49], while serum insulin levels were determined with the enzyme-linked immunosorbent assay (ELISA) using a commercial kit (DRG Diagnostics GmbH, Marburg, Germany). Serum testosterone level was measured using rat ELISA assay following the manufacturer’s instructions (DRG Diagnostics).

Acid phosphatase (ACP, EC 3.1.3.2) [50] and alkaline phosphatase (ALP, EC 3.1.3.1) [51] activities were evaluated in testicular homogenates in addition to testosterone and estradiol. Briefly, 100 mg of tissue was rinsed with 1× PBS, homogenized in 1 mL of 1× PBS, and stored overnight at −20 °C. After two freeze–thaw cycles, the homogenates were centrifuged at 5000× *g*, 4 °C for 5 min. The supernatants were collected and analyzed immediately with the ELISA kit. Protein concentrations for testicular homogenates were determined with Bradford assay for standardization of biochemical analysis [52].

### 4.10. Analysis of Antioxidant Status in Testicular Tissues

Reduced glutathione (GSH) concentration in testicular tissue was determined according to the method of Beutler et al. [53]. Lipid peroxidation was assayed with determination of malondialdehyde (MDA), the main end products of lipid peroxidation, following the protocol of Ohkawa et al. [54]. Superoxide dismutase (SOD, EC 1.15.1.1) activity was determined according to the method of Nishikimi et al. [55] depending on the ability of the enzyme to hinder the phenazine methosulphate-mediated reduction of nitroblue tetrazolium (NBT) dye. Nitric oxide (NO) level in testicular homogenate was determined as total nitrite/nitrate using the method of Montgomery and Dymock [56].

### 4.11. Western Blotting

Testicles samples were homogenized in ice-cold lysis buffer, and the homogenates were centrifuged at 14,000× *g* for 20 min at 4 °C. Samples’ protein contents were determined according to the method of Bradford (Bio-Rad Laboratories, Watford, UK) [52]. Samples of equal protein concentrations were electrophoresed using 10% SDS/PAGE and electro-transferred to polyvinylidene difluoride membranes. The membranes were blocked with 5% (*w*/*v*) skimmed milk powder in PBS/Tween-20 for 2 h at room temperature. Then, the membranes were incubated with anti-iNOS (Santa Cruz Biotechnology), anti-aromatase (diluted 1:5000), and anti-NFκB-p65 antibodies (1:250) diluted in tris-buffered saline-tween containing 1% bovine serum albumin, and β actin (Santa Cruz Biotechnology) as internal control diluted 1:1000 in blocking buffer. The membranes were incubated with the corresponding secondary antibodies for 1 h at room temperature, washed, and then developed. Protein bands were densitometrcally measured using ImageJ version 1.48 software (National Institutes of Health, Bethesda, MD, USA; http://rsb.info.nih.gov/ij/). Densities of bands were standardized to the corresponding density of β-actin.

### 4.12. Histopathology

Tissue specimens were collected from left testicles and rapidly fixed in 10% neutral buffered formalin solution for at least 24 h. Fixed samples were processed through the conventional paraffin embedding technique including dehydration through ascending grades of ethanol, clearing in three changes of xylene and melted paraffin, and ending with embedding in paraffin wax at 60 °C. Paraffin blocks were prepared, from which 3-μm-thick sections were obtained. These sections were stained with hematoxylin and eosin (H&E) [57].

### 4.13. Statistical Analysis

All data are presented as mean ± SEM. Statistical analyses were done using GraphPad Prism 5 (GraphPad Software, San Diego, CA, USA). Results were subjected to Tukey’s multiple comparisons *post-hoc*, one way ANOVA. The data serum parameters, testicular enzymes, antioxidant status, and Johnson score were statistically analyzed with one-way ANOVA followed by Duncan’s multiple range tests by the SPSS programming tool (IBM SPSS. 20^®^, IBM Corp., Armonk, NY, USA). All declarations of significance depended on *p* < 0.05.

## 5. Conclusions

Oxidative damage induced in the testicular tissues of diabetic rats is evidenced by the impairment in testicular tissues and sperm quality. Thymoquinone is considered as an excellent natural protective remedy against the inflammatory and oxidative damage caused by diabetes through upregulation of aromatase and downregulation of iNOS and NF-κB-p65 protein expression levels in testicles. Therefore, we advise enclosing thymoquinone in antidiabetic drugs.

## Figures and Tables

**Table 1:** Effect of thymoquinone (TQ) on serum glucose, insulin levels, and HA1c percentage in all experimental groups at the 12th week. Values are given as ±SEM, and Duncan’s range test ^‡^ was applied.

Experimental Group	Glucose (mg/dL)	HA1c (%)	Insulin (ng/mL)
Control	101.4 ± 2.24 ^c^	5.887 ± 0.81 ^c^	2.167 ± 0.21 ^a^
Diabetic	379.4 ± 5.04 ^a^	9.767 ± 0.46 ^a^	0.8600 ± 0.08 ^c^
Diabetic + TQ	167.6 ± 3.34 ^b^	7.100 ± 0.61 ^b^	1.733 ± 0.12 ^b^
TQ	124.8 ± 2.88 ^bc^	5.700 ± 0.30 ^c^	2.000 ± 0.05 ^b^

^‡^ SEMs bearing different superscript letters are significantly (*p* < 0.05) different from the other values within the same column. The superscript letter a is the highest mean value, then b, then c in cases of significant differences. Therefore, ^a,b,c^ in the same column are significantly different, while ^b,bc^ are non-significantly different.

**Table 2:** Effect of thymoquinone (TQ) on enzyme markers of the testicles, antioxidant status, and Johnsen score in experimental groups at the 12th week. Values are given as ±SEM, and Duncan’s range test ^‡^ was applied.

Group	Testicular Enzymes Markers		Enzymatic Status of Testicular Antioxidant				Johnsen Score
	ACP (U/mg Protein)	ALP (U/mg Protein)	MDA (nmol/mg Protein)	SOD (U/mg Protein)	GSH (nmol/mg Protein)	NO (nmol/mg Protein)	
Control	22.5 ± 2.12 ^a^	869.25 ± 6.21 ^a^	30.12 ± 4.37 ^bc^	29.2 ± 3.12 ^a^	9.12 ± 1.2 ^a^	49 ± 2.1 ^c^	9.86 ± 0.1 ^a^
Diabetic	7.8 ± 1.13 ^b^	562.12 ± 8.41 ^b^	68.15 ± 6.2 ^a^	8.9 ± 1.10 ^b^	3.22 ± 0.1 ^b^	99 ± 4.1 ^a^	4.97 ± 0.2 ^c^
Diabetic + TQ	18.2 ± 2.51 ^a^	701 ± 12.13 ^ab^	35.2 ± 5.2 ^b^	25.1 ± 3.21 ^ab^	7.25 ± 0.9 ^a^	60 ± 5.1 ^b^	8.71 ± 0.2 ^b^
TQ	23.5 ± 3.12 ^a^	889.2 ± 7.15 ^a^	25.24 ± 4.2 ^c^	30.2 ± 2.42 ^a^	9.65 ± 2.1 ^a^	48 ± 3.1 ^c^	9.77 ± 0.1 ^a^

^‡^ SEMs bearing different superscript letters are significantly (*p* < 0.05) different from the other values within the same column. The superscript letter a is the highest mean value, then b, then c in cases of significant differences. Therefore, ^a,b,c^ in the same column are significantly different, while ^a,ab^ and ^b,bc^ are non-significantly different. ACP: acid phosphatase; ALP: alkaline phosphatase; MDA: malondialdehyde; SOD: superoxide dismutase; NO: nitric oxide; GSH: reduced glutathione.

**Table 3:** Ingredients of basal diet.

Ingredients	g/kg Diet
Corn flour	529.5
Casein	200
Sucrose	100
Soybean oil	70
Cellulose	50
Mineral mix	35
Vitamin mix	10
l-cystine	3
Choline	2.5

## Abbreviations

TQ	thymoquinone
MDA	malondialdehyde
ROS	reactive oxygen species
STZ	streptozotocin
ACP	acid phosphatase
ALP	alkaline phosphatase
